# Integrated QTL detection for key breeding traits in multiple peach progenies

**DOI:** 10.1186/s12864-017-3783-6

**Published:** 2017-06-06

**Authors:** José R. Hernández Mora, Diego Micheletti, Marco Bink, Eric Van de Weg, Celia Cantín, Nelson Nazzicari, Andrea Caprera, Maria Teresa Dettori, Sabrina Micali, Elisa Banchi, José Antonio Campoy, Elisabeth Dirlewanger, Patrick Lambert, Thierry Pascal, Michela Troggio, Daniele Bassi, Laura Rossini, Ignazio Verde, Bénédicte Quilot-Turion, François Laurens, Pere Arús, Maria José Aranzana

**Affiliations:** 1 IRTA, Centre de Recerca en Agrigenòmica CSIC-IRTA-UAB-UB; Campus UAB, Bellaterra (Cerdanyola del Vallès), 08193 Barcelona, Spain; 2 Research and Innovation Centre, Fondazione Edmund Mach (FEM), Via Mach 1, 38010 San Michele all’Adige, TN Italy; 3 Hendrix Genetics Research, Technology & Services B.V., P.O. Box 114, 5830AC Boxmeer, The Netherlands; 4 Plant Breeding, Wageningen University and Research Droevendaalsesteeg 1, P.O. Box 386, 6700AJ Wageningen, The Netherlands; 5 IRTA, FruitCentreParc Cientific i Tecnològic Agroalimentari de Lleida (PCiTAL), Lleida, Spain; 6 PTP Science Park, Via Einstein, 26900 Lodi, Italy; 7 Council for Agricultural Research and Economics (CREA) Research Centre for Fodder Crops and Dairy Productions, Lodi, Italy; 8 Consiglio per la Ricerca in Agricoltura e L’analisi Dell’economia Agraria (CREA) - Centro di Ricerca per la Frutticoltura, 00134 Roma, Italy; 9 BFP, INRA, 33140 Villenave d’Ornon, France; 10 GAFL, INRA, 84140 Montfavet, France; 11 Università degli Studi di Milano, DiSAA, Via Celoria 2, 20133 Milan, Italy; 12 IRHS, INRA, SFR 4207 QuaSaV, 49071 Beaucouze, France

**Keywords:** Peach QTL, Pedigre-based Analysis, PBA, FlexQTL^TM^, Peach breeding

## Abstract

**Background:**

Peach (*Prunus persica* (L.) Batsch) is a major temperate fruit crop with an intense breeding activity. Breeding is facilitated by knowledge of the inheritance of the key traits that are often of a quantitative nature. QTLs have traditionally been studied using the phenotype of a single progeny (usually a full-sib progeny) and the correlation with a set of markers covering its genome. This approach has allowed the identification of various genes and QTLs but is limited by the small numbers of individuals used and by the narrow transect of the variability analyzed. In this article we propose the use of a multi-progeny mapping strategy that used pedigree information and Bayesian approaches that supports a more precise and complete survey of the available genetic variability.

**Results:**

Seven key agronomic characters (data from 1 to 3 years) were analyzed in 18 progenies from crosses between occidental commercial genotypes and various exotic lines including accessions of other *Prunus* species. A total of 1467 plants from these progenies were genotyped with a 9 k SNP array. Forty-seven QTLs were identified, 22 coinciding with major genes and QTLs that have been consistently found in the same populations when studied individually and 25 were new. A substantial part of the QTLs observed (47%) would not have been detected in crosses between only commercial materials, showing the high value of exotic lines as a source of novel alleles for the commercial gene pool. Our strategy also provided estimations on the narrow sense heritability of each character, and the estimation of the QTL genotypes of each parent for the different QTLs and their breeding value.

**Conclusions:**

The integrated strategy used provides a broader and more accurate picture of the variability available for peach breeding with the identification of many new QTLs, information on the sources of the alleles of interest and the breeding values of the potential donors of such valuable alleles. These results are first-hand information for breeders and a step forward towards the implementation of DNA-informed strategies to facilitate selection of new cultivars with improved productivity and quality.

**Electronic supplementary material:**

The online version of this article (doi:10.1186/s12864-017-3783-6) contains supplementary material, which is available to authorized users.

## Background

Peach [*Prunus persica* (L.) Batsch] is a fruit tree species with a relatively simple genome: diploid (2n = *2x* = *16*), small (~230 Mbp) and without any recent duplications [1]. These characteristics, together with a relatively short juvenile period (2–4 years) and a self-compatible mating system, make peach one of the model species for the Rosaceae [2, 3]. From the economic standpoint, peach production exceeds twenty million tons of fruit per year, being in the top ten of the most produced fruits worldwide (http://faostat.fao.org/). Consequently, the improvement of some of its key traits, such as those related with its production season, and pre and postharvest fruit quality, can have a major impact for the fruit industry.

Self-compatibility, selection during domestication and migration of peaches from their center of origin in China to Western Europe, and bottlenecks occurring during modern breeding, have resulted in a very narrow level of genetic variability available to breeders [1, 4, 5]. This limits the progress of breeding and makes it difficult to overcome some of the main challenges for the improvement of *P. persica*. Although most breeding programs still depend on very limited variability, some breeding initiatives have started to use genetically distant landraces or even related *Prunus* species in order to introgress specific characteristics into the current commercial materials, such as disease resistance, climate adaptation and fruit quality [6–8].

Peach consumer acceptance mostly depends on fruit quality traits such as flavor, color and size, while growers and retailers are more interested in characters such as productivity, disease resistance, a wide choice of harvest periods and post-harvest behavior [9]. The improvement of these traits could be enhanced by the use of molecular markers, but even though many marker-trait associations have been reported [3, 10, 11], their use in peach breeding programs to select major genes and quantitative trait loci (QTL) is only in the early stages [12]. One of the main reasons is the lack of concise information on the number and position of the genes determining the inheritance of a given trait, as its detection is very often based on the analysis of a single progeny with a limited number of offspring (usually *N* = 70–120). This results in the identification of only some of the QTLs, and alleles at these QTLs, affecting the trait or in an overestimation of the effects of the QTLs identified [13, 14]. In addition, the QTLs are usually not validated in different genetic backgrounds, making it difficult to extrapolate their magnitude and robustness to different breeding programs.

QTL mapping based on the joint analysis of multiple progenies can strongly alleviate some of the drawbacks of single-progeny genetic analyses. We used a Bayesian QTL mapping method, implemented in the FlexQTL software, as recently used in apple [15–17], peach and related species [18, 19], cherry [20] and strawberry [21]. These authors focused on specific characters such as firmness, sweetness-related, bud-break and flowering time in apple, fruit size in sweet cherry and disease resistance in strawberry, or on several fruit quality and productivity characters in peach. These peach studies included a relatively small number of individuals either from crosses with almond and other *Prunus* species [18] or seedlings exclusively from commercial breeding programs [19]. In this paper we analyzed a large collection (1467 seedlings) from 18 peach progenies, 17 full-sib and one half-sib families, of different European research institutions for seven relevant traits of quantitative inheritance: flowering date (FD), maturity date (MD), fruit development period (FDP), percentage of red skin overcolor (PSC), titratable acidity (TA), soluble solid content (SSC) and weight of the whole fruit (FW). FD, MD and FDP are priority traits for extending the peach production season and to adapt peaches to a changing climate, whereas PSC, TA, SSC and FW are among the most relevant traits for consumer acceptance. The set of parents used to generate these populations included a combination of elite commercial materials, landraces and peach-related species (*P. dulcis* and *P. davidiana*), allowing us to explore a large fraction of the genetic variability of the expanded *P. persica* gene pool. Our results hold promise for the identification of valuable new genes to produce a new wave of more interesting varieties for growers, retailers and consumers.

## Methods

### Plant material

The plant material used consisted of 1467 individuals from 18 progenies of five European breeding programs. These were located in INRA-Avignon (France), INRA-Bordeaux (France), IRTA-Lleida (Spain), MAS.PES program (a joint project between UMIL-Milan and CRP-Cesena, Italy) and CREA-Rome (Italy), with no duplicated individuals or common cultivars between orchards. Among them, ten were F1, five F2, two BC1 and one BC2. All of them are full-sib progenies with the exception of BC2 that is a half-sib progeny [22]. Progeny sizes ranged from 20 to 141 (Table 1). Thirteen of the progenies were derived from intra-specific crosses between peach varieties, while the other five were obtained by interspecific crosses between peach and related species, i.e., almond (*P. dulcis*) and the wild, but closely related *Prunus davidiana*. The peach parents included commercial cultivars but also non-commercial accessions, as is the case of Ferganensis peach, the ornamental ‘Weeping Flower’, the rootstocks ‘Rubira®’ and ‘Pamirskij 5’ and the cultivar ‘Bolinha’. The 11 progenies involving any of these cultivars and/or the related *Prunus* species were denominated ‘non-commercial’ (NC) progenies (see Table 1).

**Table 1:** Description of the 18 progenies included in the analysis

Cross (parents)	Acronym	Location	Commercial (C)/Non-commercial (NC) Cross^a^ type	Type of progeny	Progeny size	Measured phenotypes evaluated (number of years with evaluation available) ^b^	Reference
‘Bolero’ × ‘Oro A’	B × O	UMIL - Milan	C.	F1	72	MD(2), PSC(3), TA(2), SSC(2), FW(2)	[40]
‘Contender’ × ‘Elegant Lady’	C × EL	UMIL - Milan	C.	F1	74	MD(3)	[56]
‘Max 10’ × ‘Rebus 028’	M × R028	UMIL - Milan	C.	F1	68	MD(2), PSC(2), TA(3), SSC(3), FW(3)	-
‘Belbinette’ × ‘Nectalady’	Bb × Nl	IRTA - Lleida	C.	F1	98	FD(2), MD(3), FDP(1), TA(3), SSC(3), FW(3)	[46]
‘Big Top’ × ‘Nectaross’	Bt × Nr	IRTA - Lleida	C.	F1	44	FD(2), MD(4), FDP(2), TA(3), SSC(3), FW(3)	-
‘Big Top’ × ‘Armking’	Bt × Ak	IRTA - Lleida	C.	F1	75	FD(4), MD(4), FDP(4), TA(3), SSC(3), FW(3)	-
‘Rome Star’ × ‘BC1.25’	RS × 25	CREA - Rome	NC.	F1	20	FD(2), MD(3), FDP(2), PSC(3), TA(3), SSC(3), FW(3)	-
‘Rome Star’ × ‘BC1.61’	RS × 61	CREA - Rome	NC.	F1	25	FD(2), MD(3), FDP(2), PSC(3), TA(3), SSC(3), FW(3)	-
‘Bolinha’ × ‘Bolinha’	Bo × Bo	INRA - Avignon	NC.	F2	112	FD(2), MD(2), FDP(2), FW(2)	-
‘Ferjalou Jalousia’ × ‘Fantasia’	J × F	INRA - Bordeaux	C.	F2	141	FD(2), MD(2), FDP(2), PSC(2), TA(2), SSC(5), FW(2)	[57]
‘Weeping Flower Peach’ × ‘Pamirskij 5’	WF × P	INRA - Avignon	NC.	F2	96	FD(1), MD(1), FDP(1), TA(1), SSC(1)	-
‘Pamirskij 5’ × ‘Rubira’	P × R	INRA - Avignon	NC.	F2	96	FD(1), MD(3), FDP(1), PSC(3), TA(3), SSC(3)	[58]
‘IF7310828’ × (‘IF7310828’ x Ferganensis)	PxF	CREA - Rome	NC.	BC1	95	FD(7), MD(7), FDP(7), PSC(4), TA(4), SSC(4), FW(6)	[59]
‘Rubira’ × *P. davidiana* ‘P1908’	R × D	INRA - Avignon	NC.	F1	95	FD(1), MD(1), FDP(1), PSC(1), SSC(1)	[60]
‘Summergrand’ × *P. davidiana* ‘P1908’	SxD	INRA - Avignon	NC.	F1	67	FD(1), MD(1), FDP(1), PSC(1)	[61]
‘Texas’ × ‘Earlygold’ (‘MB1.37’)	T × E	IRTA - Lleida	NC.	F2	55	FD(2), MD(3), FDP(2), TA(3), SSC(3), FW(3)	[62]
‘MB1.37’x’Earlygold’	T1E	IRTA - Lleida	NC.	BC1	105	FD(2), MD(3), FDP(2), TA(3), SSC(3), FW(3)	[26]
‘Zephyr’ × (‘Summergrand’ × *P. davidiana* ‘P1908’)	BC2	INRA - Avignon	NC.	BC2	129	FD(6), MD(7), FDP(5), PSC(4), TA(2), SSC(5), FW(6)	[55]

^a^Those progenies derived exclusively from commercial parents were classified as commercial progenies (C) in contrast with the rest classified as non-commercial (NC) progenies

^b^Phenotypes measured for each progeny: beginning of flowering time (FD), beginning of ripening time (MD), fruit development period (FDP), percentage of red overcolor on the fruit skin (PSC), titratable acidity of the fruit flesh (TA), soluble solid content of the fruit flesh (SSC) and weight of the whole fruit (FW). The number of years for which each trait was evaluated is indicated in parentheses

### Phenotypic data

Phenotypic data for agronomic traits measured over several years were available at each location. We gathered existing data for seven of these quantitative traits: onset of flowering time (FD, date when 2–3% of flowers observed in F stage), onset of ripening time (MD, date when 2–3% of fruits were mature), fruit development period (FDP, number of days between FD and MD), percentage of red overcolor on the fruit skin (PSC, by visual estimation of the surface covered), titratable acidity of the fruit flesh (TA, meq/100 ml in the juice of at least five ripe fruits), soluble solid content of fruit flesh (SSC, average brix degrees measured in a drop of at least five ripe fruits) and fruit weight measured as the average of 10 random peaches sampled from each tree (FW, grams). The traits and number of years of observations varied per progeny (Table 1). When available, observations for two or more years were compared and analyzed for consistency; those ranking the individuals in ordinal positions differing by more than 50% between years were removed. Following, outlying observations within progenies and years were identified and removed as described by [23]. Subsequently, the year with more observations per trait and progeny was selected for the analysis. A correlation analysis was conducted to evaluate the representativeness of the selected data. In general correlations were moderate (average *r* = 0.72, median *r* = 0.76) (Additional file 1). Data for most of the traits followed a normal distribution (Additional file 2), with moderate skewness (skew ≤ 0.5), with only two of them (FD and TA) deviating from normality with positive skew.

To evaluate the environment effect introduced in the analysis by combining data from different locations, we also conducted the analysis after standardizing the raw observations following two statistical methods. In both cases the skewness of all distributions was reduced. Standardizations consisted of: i) normalizing data from the same location and year to a range between 0 and 1 (designated STD1) and ii) standardizing data from the same location and year to a variable range, with mean equal to 0 and standard deviation equal to 1 (designated STD2).

$$ S T D1=\frac{X_i-{X}_{Min}}{X_{Max}-{X}_{Min}};\kern0.75em  S T D2=\frac{X_i - {\overline{X}}_S}{\sigma_{X, S}} $$

Where ${X}_i$ is the i-th data, ${X}_{Min}$, ${X}_{Max}$ and ${\overline{X}}_S$ are the minimum, the maximum and the average of the sample data for a specific trait and ${\sigma}_{X, S}$ is the sample standard deviation.

### DNA extraction and genotyping

Genomic DNA of parents and seedlings was extracted from young leaves using the Qiagen DNeasy 96 or Mini Plant Kit® (Quiagen, MD, USA), according to the protocol provided by the supplier DNA quantification was performed for each sample using Quant-iT™ Picogreen® reagent (Invitrogen Ltd, Paisley, UK) and genotyped with the 9 K International Peach SNP Consortium array, containing 8144 SNPs scattered over the eight peach chromosomes [24]. SNPs were ordered according to their coordinates on the second version of the Peach Genome v2.0 (Peach v2.0 [25], available on GDR at https://www.rosaceae.org/species/prunus_persica/genome_v2.0.a1). Genetic positions were estimated by dividing the coordinates of the Peach Genome by the genetic length of each chromosome using the published TxE map as reference [26], which resulted in a genome-wide mean of 478.1 Kbp/cM.

The raw genotypes obtained from GenomeStudio® software were filtered using a pre-release of ASSIsT [27]. Monomorphic SNPs in all progenies or with null alleles were discarded (3142 out of 8144). The SNPs that passed the filters were grouped in haploblocks (HBs) of 1 cM, and haplotyped with PediHaplotyper [28] to reduce the computation time of the analysis and to facilitate visual inspection of markers across pedigrees.

### QTL mapping methodology

FlexQTL^TM^ software was used for the QTL analysis [15] (https://www.wur.nl/en/show/FlexQTL.htm). FlexQTL^TM^ uses bi-allelic QTLs model that allow three different genotypes (*QQ*, *Qq*, and *qq* whereby *Q* associates with high phenotypic values), and a continuous uniform distribution to assign prior QTL positions along the genome and a 2 cM binning of the genome. In this study we considered only additive effects with normal prior distribution. The models analyzed were set with a prior mean number of 15 QTLs and maximum of 20 QTLs, with the exception of the FD trait, where the mean and maximum number of prior QTLs was 25 and 40, respectively.

Additionally, “year and location” and “maturity date” were included in the model as nuisance variables, considered to follow either uniform (for the nominal “year and location” variables) or normal (for “maturity date”) distribution.

FlexQTL^TM^ was run with simulation length of 1,000,000 iterations, allowed to stop once convergence was reached after a minimum of 250,000 iterations, with a simulation chain length of at least 100 effective chain samples for the overall mean, the residual variance, the number of QTLs and the variance of such number [29, 30]. To reduce auto-correlation among samples, only one every 200 iterations was stored for further posterior inferences.

QTL evidence was estimated as in [31] using twice the natural log of the Bayes factors (2lnBF) obtained after pair-wise comparison of models differing in one QTL: 2lnBF values between two and five indicated positive evidence, between five and ten strong, and above ten decisive. Posterior QTL intensities were used to define their position on the linkage groups, as in [32], and their contributions to the observed phenotypes were obtained from the mean estimates of the QTL effect sizes. Genome-wide bin-wise breeding values (GBV) were predicted by using posterior probabilities of QTL genotypes, intensities and effect sizes while taking into account all the genome binds and considering only additive effects. All estimations and predictions were carried out using the FlexQTL^TM^ software as technically explained in [15, 33–35]. Correlation between the QTL-based genomic breeding values and the observed phenotypes in all the plant materials was used to calculate the accuracy.

All analyses were repeated at least twice (always with different seed values) in order to verify consistency of the results.

## Results

### SNP genotypes

Individuals were genotyped with the 9 K International Peach SNP Consortium array [24]. After filtering for quality, the total number of informative SNPs across all progenies was reduced from 8144 to 5002 (61.4%). The average number of informative SNPs per progeny was 2166, ranging from 349 (in BoxBo F1 progeny) to 3434 (in PxF backcross) (Additional file 3). Polymorphic SNPs mapped unevenly within and across chromosomes, revealing large homozygous regions. For example no SNP segregated in chromosome eight of the JxF progeny. Similarly, none of the SNPs of chromosomes two, four, five, seven or eight segregated in BoxBo. Closely linked SNPs were grouped in 222 haploblocks with an average position of one every 972.5 Kb. This strategy converted bi-allelic SNPs into multi-allelic HB-markers, decreasing computation time and memory requirements.

### QTL detection: number, position and additive effects, genotype probabilities, breeding values and description accuracy

**Fig. 1:**
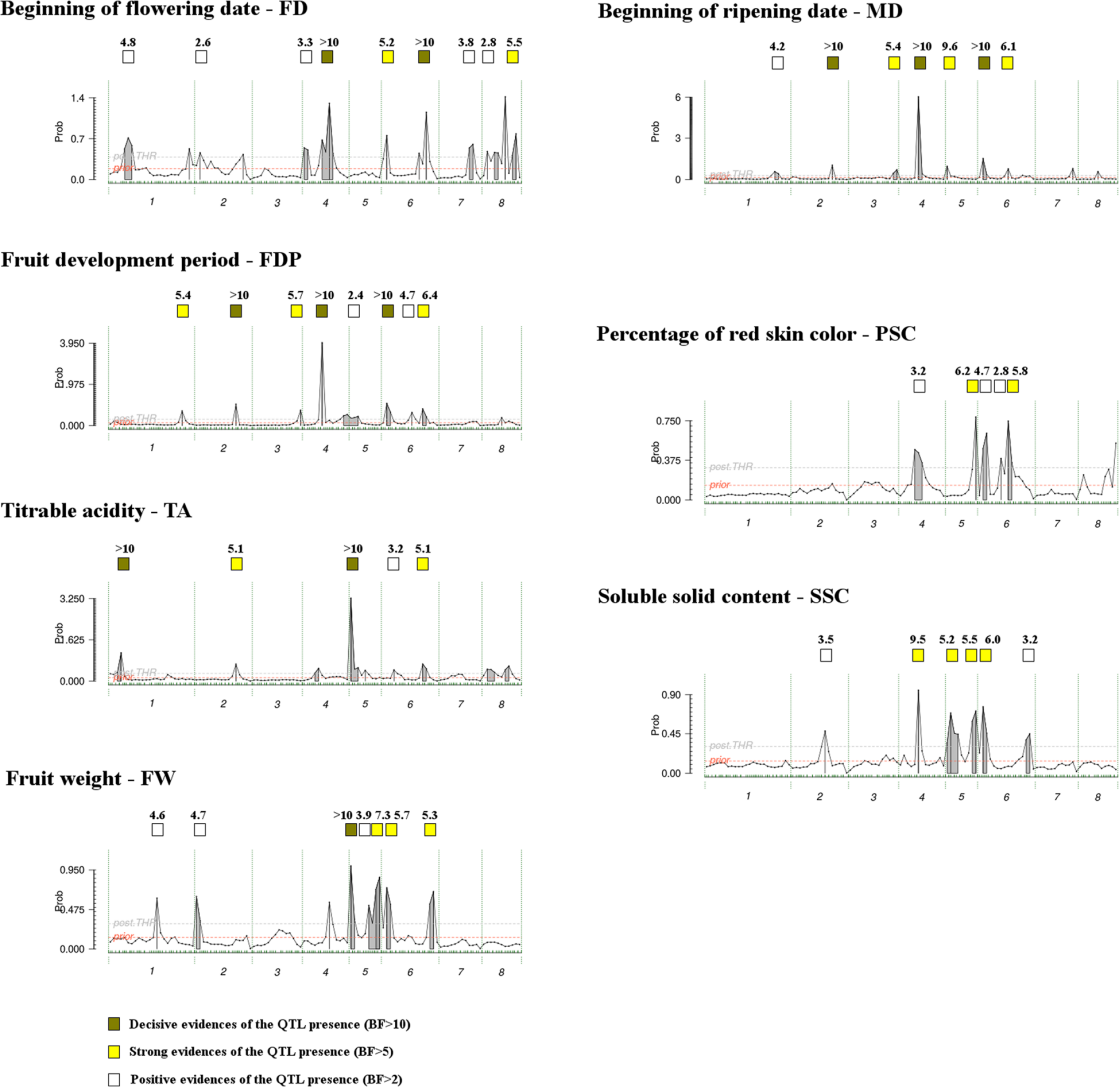
Posterior QTL models obtained with the combined original data of all progenies. *Horizontal axes* represent the eight chromosomes of the peach genome; *green ticks* indicate the SNP haplotype position and chromosomes are delimited by *dotted vertical lines. Red and gray dashed horizontal lines* indicate the bin-wise prior and posterior probability thresholds, respectively. The *filled gray areas* on the QTL peaks correspond to those bin regions exceeding the posterior bin-wise threshold probabilities. *Green, yellow and white squares* indicate the intensity of evidence (2lnBF) of the corresponding QTL

**Fig. 2:**
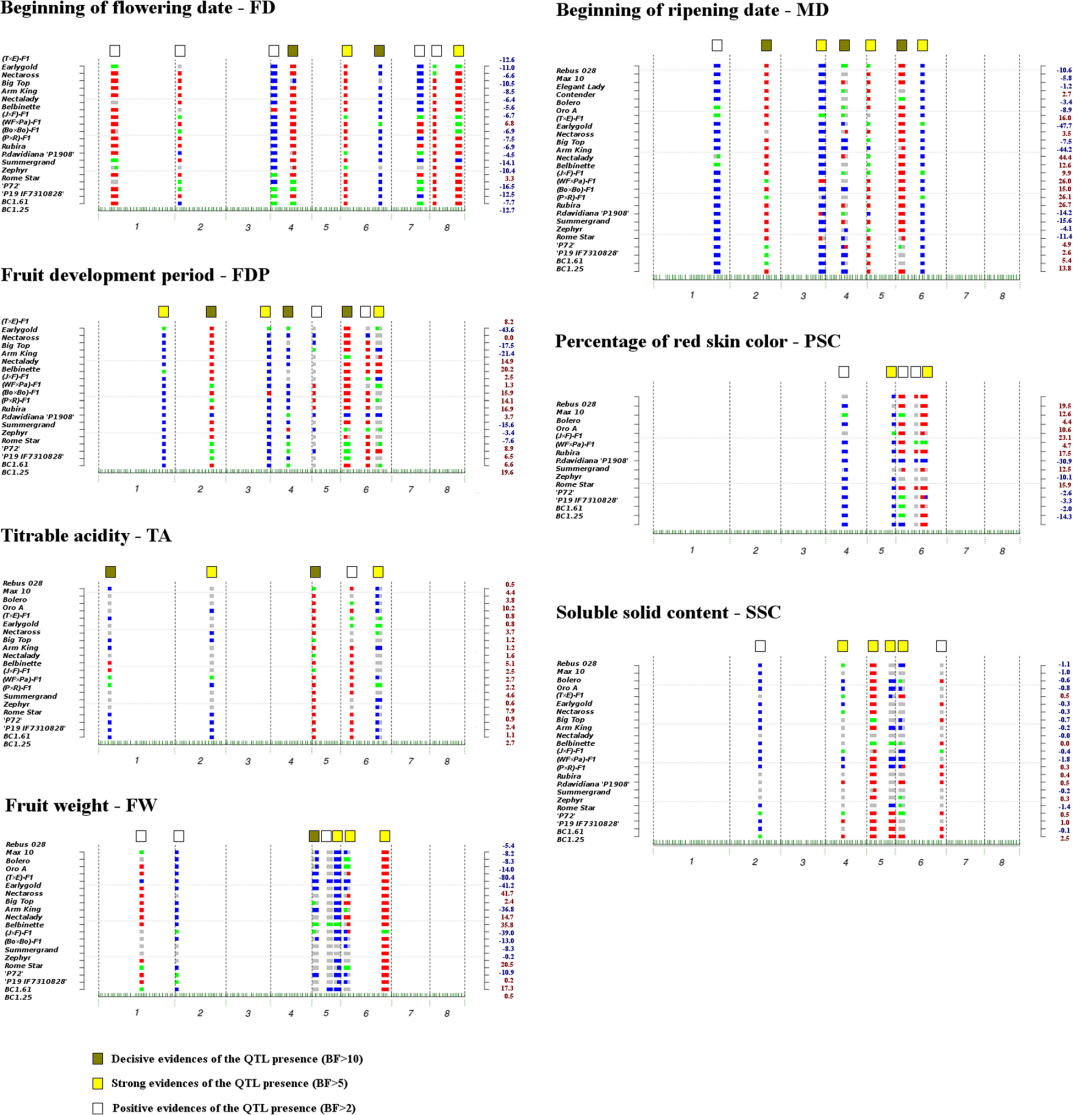
Parental line genotypes at each QTL and their genomic breeding values (GVB) estimated by FlexQTL. Genotypes for each parent are represented as *colored rectangles* at each QTL position: *blue, green and red* corresponds to *qq*, *Qq* and *QQ* genotypes respectively; while *gray* indicates unclear genotype possibilities. GBV for each parental line are indicated at the *right axes* of each plot with *blue or red numbers* depending on their contribution to decreasing or increasing the phenotypic value among their progenies, respectively

FlexQTL^TM^ software was used to detect QTLs for each of the seven traits by conducting an integrated analysis of phenotypic and genetic data of all progenies. The number of individuals analyzed per trait varied between 570 for red skin coloration (PSC) to more than 1160 for the QTLs responsible for FD and MD. Analyses were performed with original and both standardized observations (STD1 and STD2). The estimated narrow-sense heritability of the traits (h^2^) was lower with the original data than after standardization which brought h^2^ up to 0.63 for SSC to 0.94 for MD, with an average of 0.83. All putative QTLs detected with at least positive evidence (2lnBF > 2) with the original data and in one or both standardizations; or those with decisive evidence (2lnBF > 10) in one of the three data sets were considered for further examination and discussion (Fig. 1, Table 2). Overall the analysis identified 47 QTLs meeting this requirement (nine for FD, eight for FDP, seven for MD and FW, six for SSC and five for PSC and TA). Most of the QTLs (30) had high evidence: 11 were decisive (2lnBF > 10) and 19 strong (5 < 2lnBF < 10). Confidence intervals ranged from 0.21 Mb to 4.71 Mb, with an average of 1.85 Mb. Thanks to the QTL genotypes estimated by FlexQTL per parental line (Fig. 2), the progenies contributing to each of the detected QTL could be identified. For 38 QTLs, the genotype for each parent was assigned as *qq*, *Qq* or *QQ* (represented in Fig. 2 in blue, green and red, respectively), while for ten QTLs (qFDP5, qFDP6.2, qTA1, qTA2, qSSC4, qSSC5.2, qSSC6.1, qSSC6.2, qFW5.1 and qFW5.2) no genotypes could be assigned for more than 25% of the parents. Figure 2 also shows the estimated genome-wide, bin-wise breeding values (GBV) of the parents, indicating their contribution in decreasing (blue numbers) or increasing (red numbers) phenotypic values among their progenies. Most of the parents had a genetic potential in advancing flowering but not in anticipating maturity date. Similarly, all showed a certain capacity for increasing TA levels.

**Table 2:** Heritability, intervals, additive effect and evidences of the QTLs identified with FlexQTL software

TRAIT (sample size)^a^	Heritability (h^2^)	QTL name^b^	Involved haploblocks^c^	Position interval^d^	Additive effect	Evidences (2lnBF)^e^	CO^f^	NUI1^g^	NUI2^h^
FD (1163)	0.92	qFD1	1.09–1.13	9,262,115–13,801,920	6.9	positive (4.8)		x	
		qFD2	2.03	3,205,511–4,172,142	1.2	positive (2.6)	x	x	
		qFD4.1	4.01–4.03	1,117,074–4,056,544	3.6	positive (3.3)	x	x	
		qFD4.2	4.13–4.15	13,123,062–16,084,695	12.7	decisive (>10)		x	
		qFD6.1	6.03	3,163,789–4,096,358	2.1	strong (5.2)	x	x	
		qFD6.2	6.25	25,235,588–26,118,990	4.9	decisive (>10)		x	
		qFD7	7.17–7.19	17,205,368–20,002,222	2.7	positive (3.8)	x	x	
		qFD8.1	8.03	3,177,246–3,925,327	1.4	positive (2.8)		x	
		qFD8.2	8.17–8.19	17,207,719–20,084,244	4.9	strong (5.5)		x	
MD (1166)	0.94	qMD1	1.39–1.41	40,030,681–41,980,791	16.8	positive (4.2)		x	
		qMD2	2.23	23,368,508–24,174,472	4.9	decisive (>10)		x	
		qMD3	3.25–3.27	25,062,869–27,310,140	20.1	strong (5.4)		x	
		qMD4	4.11–4.13	11,208,348–14,108,774	15.6	decisive (>10)	x	x	
		qMD5	5.01	1,376,476–2,240,658	5.4	strong (9.6)	x	x	
		qMD6.1	6.03–6.05	3,163,789–6,049,306	10.4	decisive (>10)	x	x	
		qMD6.2	6.17	17,252,031–18,109,410	5.7	strong (6.1)	x	x	
FDP (966)	0.92	qFDP1	1.41	41,413,143–41,980,791	9.8	strong (5.4)		x	
		qFDP2	2.23	23,368,508–24,174,472	5.3	decisive (>10)		x	
		qFDP3	3.27	27,096,340–27,310,140	10.6	strong (5.7)		x	
		qFDP4	4.11	11,208,348–12,107,192	10.0	decisive (>10)	x	x	
		qFDP5	5.01	1,376,476–2,240,658	4.3	positive (2.4)	x	x	
		qFDP6.1	6.03–6.05	3,163,789–6,049,306	11.7	decisive (>10)	x	x	
		qFDP6.2	6.17	17,252,031–18,109,410	5.1	positive (4.7)	x	x	
		qFDP6.3	6.23–6.25	23,319,780–26,118,990	8.0	strong (6.4)		x	
PSC (570)	0.71	qPSC4	4.11–4.13	11,208,348–14,108,774	23.6	positive (3.2)	x	x	
		qPSC5	5.17	17,920,002–18,236,498	13.0	strong (6.2)	x	x	x
		qPSC6.1	6.03–6.05	3,163,789–4,096,358	30.8	positive (4.7)	x	x	x
		qPSC6.2	6.13	13,165,672–13,743,179	9.2	positive (2.8)			x
		qPSC6.3	6.17–6.19	17,252,031–20,094,864	26.0	strong (5.8)		x	x
TA (818)	0.90	qTA1	1.07	7,374,062–7,949,419	3.5	decisive (>10)		x	x
		qTA2	2.23	23,368,508–24,174,472	1.3	strong (5.1)		x	x
		qTA5	5.01	1,376,476–2,240,658	7.7	decisive (>10)	x	x	x
		qTA6.1	6.07	7,550,351–8,127,200	1.4	positive (3.2)		x	
		qTA6.2	6.23–6.25	23,319,780–26,118,990	3.4	strong (5.1)		x	x
SSC (855)	0.63	qSSC2	2.19	19,233,501–20,101,839	0.7	positive (3.5)		x	x
		qSSC4	4.11	11,208,348–12,107,192	0.9	strong (9.5)	x	x	x
		qSSC5.1	5.01–5.05	1,376,476–6,071,714	3.2	strong (5.2)	x	x	x
		qSSC5.2	5.15–5.17	15,249,345–18,236,498	1.5	strong (5.5)	x	x	x
		qSSC6.1	6.03–6.05	3,163,789–6,049,306	1.9	strong (6.0)	x	x	x
		qSSC6.2	6.29	29,231,387–30,104,758	1.2	positive (3.2)		x	x
FW (777)	0.78	qFW1	1.27	27,194,373–28,053,064	15.1	positive (4.6)		x	x
		qFW2	2.01–2.03	1,197,660–4,172,142	29.7	positive (4.7)	x	x	x
		qFW5.1	5.01–5.03	1,376,476–4,224,621	38.7	decisive (>10)	x	x	x
		qFW5.2	5.11–5.13	11,258,684–14,161,849	36.0	positive (3.9)		x	x
		qFW5.3	5.15–5.17	15,249,345–18,236,498	73.8	strong (7.3)	x	x	x
		qFW6.1	6.03–6.05	3,163,789–6,049,306	44.2	strong (5.7)	x	x	x
		qFW6.2	6.27–6.29	27,164,548–30,104,758	78.0	strong (5.3)	x	x	x

^a^FD = flowering time; MD = onset of ripening (MD); FDP = fruit development period, PSC = percentage of red overcolor on the fruit skin, TA = titratable acidity of the fruit flesh, SSC = soluble solid content of the fruit flesh, FW = fruit weight

^b^QTLs were named as follows: q + trait name + chromosome + ordinal position of the QTL in the chromosome (when more than one) (i.e. “qFD4.1” refers to the first QTL for FD in chromosome 4)

^c^Haploblocks are designated by the chromosome number followed by the physical position (in Mb, according to Peach v2.0) of the first SNP of the block (i.e. “1.09” indicates that the first SNP of the haplotype involved in the QTL is in the 9 Mb region of chromosome 1)

^d^The interval position corresponds to the peak areas over the posterior bin-wise threshold probabilities

^e^ Evidences, measured as the 2lnBF, calculated on the original (raw) phenotypic data

^f^QTLs identified in the analysis of commercial progenies only

^g^Phenology and fruit quality QTLs using the “year of evaluation” and the “location” as nuisance variable (NUI1)

^h^Fruit quality QTLs using maturity date (MD) as nuisance variable (NUI2)

The stability of fruit quality QTLs was checked by adding two nuisance variables to the FlexQTL analysis: the orchard location and evaluation year (NUI1) and the maturity date (NUI2). When adding NUI1 in the analysis, all but one of the three QTLs for the PSC trait in chromosome 6 (qPSC6.2) were detected (Table 2). Similarly, all fruit trait QTLs except qPSC4 and qTA6.1 were detected when including the NUI2 in the analysis. In all cases, the missing QTLs had previously shown low evidence (2lnBF equal to 2.8, 3.2 and 3.2, respectively) as well as low additive effect.

Seven of the families analyzed derived from crosses between commercial or improved varieties, while the remaining derived from non-commercial or non-persica parents. To enhance detection of the QTLs putatively more representative in commercial germplasm, we performed the analysis using only the progenies of the commercial parents. In total we detected 25 QTLs, all already detected when analyzing together all progenies. Among them, five were for FW, four for FD, MD, FDP and SSC, three for PSC and one for TA (Table 2).

Accuracy was estimated through the correlations between the progeny phenotypes and their estimated QTL-based GBV obtained with original data as well as with standardized measures (Table 3).

**Table 3:** Accuracies with original (raw) and standardized data (STD1 and STD2)

Progenies	FD^a^			MD^b^			FDP^c^			PSC^e^			TA^e^			SSC^f^			FW^g^		
	raw	STD1	STD2	raw	STD1	STD2	raw	STD1	STD2	raw	STD1	STD2	raw	STD1	STD2	raw	STD1	STD2	raw	STD1	STD2
BxO	-	-	-	0.91	0.91	0.87	-	-	-	0.76	0.82	0.80	0.69	0.85	0.85	0.78	0.80	0.81	0.87	0.90	0.90
CxEL	-	-	-	0.88	0.90	0.89	-	-	-	-	-	-	-	-	-	-	-	-	-	-	-
MxR028	-	-	-	0.97	0.97	0.96	-	-	-	0.69	0.75	0.73	0.94	0.94	0.93	0.87	0.82	0.84	0.86	0.88	0.90
BbxNl	0.95	0.96	0.97	0.95	0.86	0.84	0.93	0.78	0.74	-	-	-	0.98	0.96	0.92	0.89	0.88	0.85	0.93	0.91	0.91
BtxNr	0.79	0.71	0.72	0.94	0.93	0.92	0.95	0.86	0.85	-	-	-	0.89	0.82	0.82	0.82	0.77	0.75	0.70	0.61	0.64
BtxAk	0.49	0.53	0.45	0.80	0.93	0.88	0.82	0.86	0.84	-	-	-	0.88	0.83	0.80	0.69	0.68	0.70	0.79	0.81	0.84
RSx25	0.94	0.95	0.93	0.40	0.84	0.71	0.33	0.92	0.60	0.40	0.87	0.88	0.71	0.92	0.65	0.67	0.92	0.51	0.89	0.91	0.92
RSx61	0.89	0.92	0.90	0.88	0.92	0.85	0.90	0.96	0.91	0.58	0.74	0.63	0.69	0.92	0.71	0.38	0.92	0.65	0.85	0.89	0.88
BoxBo	1.00	0.83	0.71	0.58	0.62	0.63	0.58	0.51	0.55	-	-	-	-	-	-	-	-	-	0.92	0.99	0.99
JxF	0.96	0.92	0.87	0.93	0.87	0.88	0.92	0.89	0.88	0.71	0.67	0.68	0.94	0.81	0.80	0.65	0.61	0.62	0.85	0.87	0.89
WFxP	0.89	0.70	0.67	0.28	0.74	0.76	0.75	0.72	0.73	-	-	-	0.73	0.67	0.67	0.32	0.28	0.30	-	-	-
PxR	0.35	0.92	0.87	0.69	0.73	0.68	0.63	0.68	0.67	0.74	0.71	0.72	0.84	0.80	0.79	0.33	0.29	0.31	-	-	-
PxF	0.95	0.90	0.89	0.78	0.81	0.79	0.86	0.83	0.83	0.72	0.74	0.72	0.44	0.61	0.62	0.62	0.57	0.59	0.73	0.66	0.75
RxD	0.78	0.69	0.62	0.27	0.72	0.92	0.77	0.65	0.64	0.66	0.61	0.52	-	-	-	0.94	0.73	0.65	-	-	-
SxD	0.76	0.91	0.84	0.84	0.85	0.82	0.81	0.78	0.80	0.77	0.80	0.76	-	-	-	-	-	-	-	-	-
TxE	0.75	0.71	0.75	0.96	0.90	0.88	0.93	0.84	0.78	-	-	-	0.69	0.65	0.54	0.76	0.78	0.80	0.22	0.25	0.30
T1E	0.59	0.58	0.50	0.97	0.90	0.88	0.93	0.76	0.72	-	-	-	0.79	0.85	0.81	0.53	0.46	0.47	0.47	0.40	0.30
BC2	0.99	0.99	0.99	0.90	0.94	0.91	0.97	0.90	0.89	0.96	0.93	0.95	0.93	0.85	0.86	0.91	0.89	0.86	0.96	0.97	0.99
All progenies	0.98	0.92	0.87	0.91	0.89	0.85	0.94	0.83	0.83	0.84	0.80	0.75	0.91	0.82	0.77	0.82	0.74	0.68	0.91	0.88	0.89

Correlation between progenies phenotypes and their estimated genome-wide breeding values (GBV) (accuracy) was obtained for ^a^flowering date (FD), ^b^maturity date (MD), ^c^fruit development period (FDP), ^d^percentage of red skin color on the fruit skin (PSC), ^e^titratable acidity of the fruit flesh (TA), ^f^soluble solid content of the fruit flesh (SSC) and ^g^weight of the whole fruit (FW))

When considering all progenies and original data, the highest accuracy was obtained for FD (0.98), followed by FDP (0.94), MD, TA and FW (all three, 0.91), and PSC (0.84) and SSC (0.82). Accuracy for SSC prediction was also the lowest for the two standardization methods (0.74 for STD1 and 0.68 for STD2). As shown, the correlation between GBV and the observed phenotypes varies for each trait and also progeny; on average, accuracies were higher for commercial progenies than those designed for experimental genetic studies.

A more detailed description of the results shown in Table 2, for each trait, is given in the following paragraphs.

### Flowering date (FD)

The narrow-sense heritability (h^2^) estimated by FlexQTL with original data was 0.92. Nine putative QTLs were identified on six chromosomes: in G1 (qFD1), G2 (qFD2), G4 (qFD4.1 and qFD4.2), G6 (qFD6.1 and qFD6.2), G7 (qFD7) and G8 (qFD8.1 and qFD8.2), with additive effects ranging from 1.2 to 12.7 days. The average interval size for FD QTLs was 2.18 Mb. The qFD4.2 and qFD6.2 QTLs were decisive while that for qFD6.1 and qFD8.2 were detected with strong evidence. The families contributing to the decisive QTL qFD4.2 were PxF (both parents with inferred QTL genotype *Qq*), RSx25 (the female parent *QQ* and the pollen parent *Qq*), and BtxNr (the female parent with estimated genotype ‘*qq*’ and the pollen parent with estimated genotype ‘*QQ*’). The only progeny that contributed to the other decisive QTL (qFD6.2) was WFxP, arosed from the selfing of a *Qq* heterozygous hybrid. These two QTLs, the strong QTL on chromosome 8 (qFD8.2), and the positive qFD1 and qFD8.1 were lost when analyzing only the commercial families, with only four of the QTLs remaining (one per chromosome 2, 4, 6 and 7). Genome-wide breeding values for each parent are shown in Fig. 2. All but two parents showed potential in advancing flowering date between 4.5 and 16.5 days. Only the hybrid ‘Weeping Flower Peach’ x ‘Pamirskij 5’ (WFxP) and the parent ‘Rome Star’ delayed flowering (by 6.8 and 3.3 days respectively). Accuracy of the predictions was in general high (0.98 in total, 0.79 in average of all progenies) with the exception of that for PxR, BtxAk and T1E. The accuracy only increased for PxR after data standardization (from 0.35 with original data to 0.92 and 0.87 with STD1 and STD2, respectively). GBVs for the BoxBo progeny correlated 100% with the observed phenotypes.

### Maturity date (MD)

The maturity date (MD) h^2^ estimated with original data was 0.94. In total, seven putative QTLs were detected for MD on six chromosomes: in G1 (qMD1), G2 (qMD2), G3 (qMD3), G4 (qMD4), G5 (qMD5) and G6 (qMD6.1 and qMD6.2), with additive effects ranging from 4.9 to 20.1 days (average 11.3 days). The average interval size for MD QTLs was 1.79 Mb, ranging from 0.81 Mb to 2.90 Mb. Three QTLs (qMD2, qMD4 and qMD6.1) were detected with decisive evidence (ln2BF >10) while those for qMD3, qMD5 and qMD6.2 had strong evidence. Two of the QTLs for which evidence was decisive (qMD4 and qMD6.1) also had a large effect (15.59 and 10.37 days respectively); for both, the confidence interval spanned close to 3 Mb. The four progenies contributing to the decisive QTL in chromosome 2 were all non-commercial (PxF, RSx61, WFxP and PxR, whereby the allele for early maturation came from P19-IF7310828, BC1.61 and ‘Pamirskij 5,’). In contrast, the QTL qMD4, also decisive, was discovered in commercial families. After removing non-commercial progenies from the analysis, QTLs on G1, G2 and G3 disappeared and only those on G4, G5 and G6 remained. The ‘Earlygold’ and ‘Armking’ varieties had the highest capacity for accelerating maturity (GBV equal to −47.7 and −44.4 days, respectively) while ‘Nectalady’ had the greatest genetic ability for delaying maturity (GBV = 44.4 days). The accuracy of predictions was 0.91 when considering all progenies original phenotypic observations. It was poor for RxD and WFxP families (0.27 and 0.28 respectively) while MxR028, T1E and TxE were the most accurate (0.97, 0.97 and 0.96, respectively). In general accuracies improved with both standardizations, especially with STD1.

### Fruit Development Period (FDP)

The fruit development period was obtained by subtracting the flowering date from the maturity date. For this trait, FlexQTL estimated h^2^ = 0.92. The QTLs identified coincided with those for MD with one additional QTL in G6 (qFDP6.3) with strong evidence (2lnBF = 6.4) and high effect (8 days). As for MD, evidence for qFDP4 and qFDP6.1 were decisive and had high effect on the phenotype (9.97 and 11.74 days, respectively). On average, the intervals for the eight QTLs were narrower than those established for MD and FD, ranging from 0.21 Mb to 2.89 Mb and an average of 1.23 Mb. As for MD, the decisive QTL qFDP2 was generated by progenies containing ‘Pamirskij 5’ and Ferganensis, and disappeared when removing non-commercial progenies from the analysis. ‘Belbinette’ and ‘BC1.25’ had higher positive GBV (20.2 and 19.6 days, respectively), while the highest ability in shortening fruit development period was assigned to ‘Earlygold’ (GBV = −43.6 days). The overall GBV of progenies correlated well with observations. When considering all progenies, data correlations were 0.94. The progeny for which prediction accuracy was lowest was RSx25 (0.33 for raw data, 0.92 for STD1 and 0.6 for STD2).

### Percentage of skin overcolor (PSC)

For PSC, FlexQTL estimated h^2^ = 0.71 and identified five putative QTLs on three chromosomes, one in G4 (qPSC4), one in G5 (qPSC5) and three in G6 (qPSC6.1, qPSC6.2, qPSC6.3). None was identified with decisive evidence. There was strong evidence for qPSC5 and qPSC6 and positive for the three remaining (qPSC4, qPSC6.1 and qPSC6.2). Additive effects ranged from 9.2 to 30.8% of red skin overcolor (20.5% on average). QTL intervals covered an average of 1.51 Mb; the QTL qPSC5 (with strong evidence and high effect) was the one detected with the narrowest interval (0.32 Mb), while the other strong QTL (qPSC4) covered a much larger region (2.90 Mb). The strong QTL in chromosome 5 was identified in the progeny JxF, while WFxP and PxF segregated for qPSC6.3 where the non-commercial line ‘Pamirskij 5’ provided the rare allele for low PSC. The *Qq* x *qq* BxO progeny contributed to qPSC4. Ferganensis, *P. davidiana* or ‘Zephyr’ contributed negatively to the PSC trait, while the F1 hybrid of ‘Ferjalou Jalousia’ x ‘Fantasia’ (JxF) contributed most to red skin coverage. In general, commercial parents contributed positively to increase the percentage of skin overcolor (average of GBV = 11.06) while non-bred parents contributed to reducing skin overcolor (average GBV = −4.41). The correlation between GBV and the percentage of skin red overcolor observed was lower than that of the previous traits (0.84). As for FDP, the prediction for RSx25 was low (0.4) for the original data but improved after data standardization (data not shown).

### Titratable acidity (TA)

Narrow-sense heritability for TA was 0.90. Five putative QTLs were identified on four chromosomes, one in G1, one in G2, one in G5, and two in G6 (qTA6.1 and qTA6.2). Two had decisive evidence (qTA1 and qTA5) and high additive effect (3.5 and 7.7 meq/100 ml, respectively), spanning a region of 0.58 and 0.86 Mb, respectively. The average confidence interval of all the QTLs was 1.12 Mb. When considering only the parents with assigned genotype at qTA1, the progenies that led to the discovery of this QTL were WFxP and PxR. The families contributing to the QTL on chromosome 5 were MxR028, BbxNl, JxF, BtxA and BtxNr, whereas the low acid allele came from parents Rebus 028, ‘Nectalady’, ‘Big Top’ and the hybrid from ‘Ferjalou Jalousia’ and ‘Fantasia’ (Fig. 2). All parents were potentially able to increase acidity. The parents with lower GBV were the sub-acid varieties ‘Rebus 028’ and ‘Zephyr’ (0.5 and 0.6, respectively), while those increasing the acidity more were ‘Oro A’ and ‘Rome Star’ (GBV = 10.2 and 7.9, respectively).

To minimize a possible epistatic effect of the maturity date on TA content we included MD data as a nuisance variable in the genetic model. All QTLs except qTA6.1 (with low effect in the initial analyses) were maintained. The correlation between GBV and TA was high (*r* ^2^ = 0.91). The model was able to better predict the breeding value for the BbxNl (accuracy 0.98), MxR028 and JxF (both with accuracy of 0.94) progenies. Predictions were worst for PxF, although, as previously, they increased after standardizations.

### Soluble solids content (SSC)

Narrow-sense heritability for SSC was 0.63. For this trait we identified six putative QTLs on four chromosomes: on G2 (qSSC2), G4 (qSSC4), G5 (qSSC5.1 and qSSC5.2) and G6 (qSSC6.1 and qSSC6.2). QTLs were detected with either strong (qSSC4, qSSC5.1, qSSC5.2 and qSSC6.1) or positive evidence (qSSC2 and qSSC6.2). The additive effect of QTLs ranged from 0.7 °BRIX to 3.2 °BRIX and covered a region of 4.7 Mb. The model failed to assign the most probable genotype to more than 25% of the parents in qSSC4, qSSC5.2, qSSC6.1 and qSSC6.2. The families contributing to qSSC5.1 were those containing ‘Big top’ and ‘Belbinette’. The latter variety also contributed to the strong QTLs qSSC5.2 and qSSC6.1. All QTLs were maintained after including MD data as a nuisance variable, while the positive ones disappeared in the analysis including only the commercial crosses. Breeding values were positive (increased SSC) for most of the non-commercial parents and negative, although close to zero, for the commercial ones. These values showed a correlation of 0.82 with the measures obtained and, at progeny level, were similar when analyzing raw and standardized data. The average accuracy per population was 0.66, ranging from 0.32 (in WFxP) to 0.94 (in RxD), with an average value of 0.66.

### Fruit Weight (FW)

Narrow-sense heritability for fruit weight was 0.77. Seven QTLs were detected on four chromosomes: one in G1 and in G2, three in G5 (qFW5.1, qFW5.2 and qFW5.3) and two in G6 (qFW6.1 and qFW6.2). QTL evidence was decisive for the one at the top of G5 (qFW5.1) with additive effect of 38.7 g covering a region of 2.9 Mb. Strong evidence was assigned to qFW5.3, qFW6.1 and qFW6.2, with additive effects equal to 73.9, 44.2 and 78.0 g, respectively.

All QTLs were maintained after including MD as a nuisance variable in the model. The analysis with exclusively commercial progenies detected all but qFW1 and qFW5.2, both with positive evidence only on analysing the full germplasm. The first was based on the segregation in the F1 individual from the cross ‘IF7310828’ x Ferganensis (P-IFxF), and the parents ‘Rebus 028’ and BC1.25, with overall breeding values of −10.9, −5.4 and 0.5 g, respectively. Belbinette contributed to the second (GBV = 35.8 g) (Fig. 2). The parents with higher GBV were ‘Nectaross’ and ‘Belbinette’ (41.7 and 35.8 g, respectively). Correlations between GBV and weights were high, 0.91, when analyzing all progenies, and lower for TxE and T1E (0.22 and 0.47, respectively).

## Discussion

In this study, we present the results of the integrated analysis of 18 families from different European breeding programs, with the discovery and characterization of QTLs involved in seven of the most important agronomical traits in peach. A novel strategy for QTL analysis was applied: we integrated genotype data from different progenies, as well as phenotype data obtained in different orchards and years and, therefore, under different environmental conditions. The data was statistically standardized and nuisance variables were introduced to minimize the environmental effect while evaluating the stability of the QTLs. This strategy contrasts with previous methodologies using single year and single location phenotypic data or where multi-year and multi-site data were integrated through the use of common reference cultivars [15] to identify QTLs, and is in line with the use of nuisance variables to account for genetic structure in cases where the genetic links between families cannot be monitored through genotyped pedigrees [18]. Our strategy allowed a considerable increase in the sample size for the QTL analysis compared to previous studies on single families. Another new feature was to transform the bi-allelic SNP markers into multi-allelic markers by constructing SNP-haploblocks and their related haplotypes.

### Heritability & accuracy

FlexQTL integrated analysis generated models with high estimated narrow sense heritabilities (h^2^ = 0.63–0.94). The h^2^ values for MD (0.94), FDP (0.92) and PSC (0.71) were within the range of those found before by other authors using classical heritability analyses: 0.87–0.94 for MD, 0.91 for FDP and 0.68 for PSC [36–38]. For the other four characters, FD (0.92), TA (0.90), SSC (0.63) and FW (0.78), these values were close to the upper boundary of those available: 0.60–0.90 for FD, 0.31–0.96 for TA, 0.33–0.77 for SSC and 0.32–0.60 for FW [36, 38, 39]. Although the highest values for TA (0.96) and SSC (0.77) are estimates of broad sense heritability (H^2^) [39], these data are consistent with intermediate to high heritability of the characters analyzed. Our observations are in agreement with the broad sense heritabilities calculated by [18] in peach materials introgressed with almond and other related *Prunus* for six of these characters (all but PSC). H^2^ values were similar to those for h^2^ that we calculated for FD, MD and FW, and slightly lower for FDP, TA and SSC. Our higher heritability estimates may be due to the different sets of populations used (including various intraspecific peach x peach progenies in our case), the larger sample examined (1467 vs. 409 individuals), or both.

Even though the models we used only took into account the additive effects, the accuracy between explained and observed phenotypes was very high: from the highest 0.98 Pearson’s correlation for FD to the lowest 0.82 for SSC (Table 3). As expected, traits showing higher heritability (FD, MD, FDP, TA and FW) were also those with higher correlations between predicted and observed phenotypes (>0.9). In fact, as previously mentioned, the accuracy of our models was usually lower for non-commercial progenies than for those generated from crosses with only commercial peach varieties (i.e., MxR028, CxEL, BxO, BtxNr, BtxAk, BbxNl and JxF). This may be explained by the fact that fewer alleles are present in commercial varieties, and at higher frequency, so their effects are better estimated by the models we used compared to those of exotic materials, which often segregate in one or a few progenies.

Although in general accuracies were slightly higher for raw data, for some families and traits, standardizations improved accuracies up to 178% for STD1 (RSx25 from 0.33 to 0.92 for FDP) and up to 241% for STD2 (RxD from 0.27 to 0.92). In all cases these families had a reduced sample size or only one year of available observations, which prevented for quality data filtering.

### Origin of novel variability

Of the 47 putative QTLs obtained, 22 (47%) would not have been found when analyzing the subset of seven commercial progenies (Table 2), nine with positive, eight with strong and five with decisive evidence. For 7 of these (15% of the total), qFD6.2, qMD3, qFDP3, qPSC6.2, qPSC6.3, qSSC2 and qTA2, the parents carrying variant alleles were all exotic lines or hybrids between commercial and exotic genotypes (see Fig. 2), suggesting that this is a fraction of variability essentially from outside the peach commercial gene pool. On the other hand, no QTL detected with the whole set of progenies was detected exclusively when using only the set of commercial progenies (Table 2). This highlights the importance of incorporating novel variability into the peach commercial gene pool or, more specifically, increasing effective population size in the set of progenies considered. These results exemplify also that the model used was more efficient in detecting QTLs when using a larger number of populations as four of the QTLs detected with the 18 populations (qPFD1, qTA6.1, qSSC6.2 and qFW5.2) were not detected when using only the subset of the commercial peach populations, although they were expected to segregate in some of them as it can be deduced from the estimated genotypes of their parents in Fig. 2.

### Co-localization with previously described major genes and QTLs and pleiotropic effects

Some of the QTLs detected coincided with the position of major genes already reported. This is the case of qMD4 with decisive evidence, mapping to the same region in the central part of G4 as the *MD* gene determining maturity date [40, 41]. A major QTL in this region has been identified in other peach progenies as well as in crosses involving other *Prunus* crops [42].

Another major gene, *D*, responsible for the acid vs. subacid fruit taste in peach and located at the proximal end of G5 [43, 44] co-maps with a decisive QTL for TA (qTA5). The dominant allele conferring the subacid character is present in some of the commercial parents studied, e.g. ‘Big Top’, ‘Nectalady’, ‘Rebus 028’ and ‘Ferjalou Jalousia®’ (one of the grandparents of JxF progeny) (Fig. 2). This indicates that this locus may be the responsible for the low-acid boundary while the remaining QTLs may contribute to the variation on the acidity levels.

QFW6.1, a QTL with strong evidence for fruit weight, maps to the end of G6, where the *S* gene that determines the flat vs. round shape of the fruit is located [43, 45]. This QTL is heterozygous only in the JxF progeny (Fig. 2), consistent with the fact that only this progeny segregates for the fruit shape phenotype, and that flat peaches are usually lighter than the round ones.

QTL-discovery data were previously published for eight of the 18 families currently analyzed: BxO [40], BbxNl [46], PxF [47], JxF [43, 48], BC2 [22], and TxE and T1E [8]. We counted the QTLs of these publications that were consistent (found in at least two years or locations and mapping at sites compatible to those found in this study): only 22 (47%) of the 47 QTLs detected by FlexQTL had previously been identified. The remaining 25 QTLs were only present in the other ten families or segregated in these eight families but remained below the significance threshold when using a conventional bi-parental approach. Examples of the latter for strong to decisive QTLs are qFD8.2, qTA6.1, qMD3, qFDP1, qFDP3, qFDP6.3 and qSSC4 for TxE and T1E and qFD5, qFD8.2, qMD5, qPSC5 and qFW5 for JxF, where heterozygous parents are predicted by the model (see Fig. 2) but no QTL was identified in the corresponding previously published single-progeny analyses. This illustrates the two advantages of the multi-progeny approach used in this work: the analysis of a large set of individuals from various crosses improves both genetic variability considered and the power of detection of the QTLs.

Thirteen of the 30 QTLs with strong to decisive evidence are described here for the first time, taking into account all previous peach QTL studies (those mentioned in the previous paragraph plus [18, 49–53]). Five of these QTLs were identified for FDP (qFDP1, qFDP2, qFDP3, qFDP6.1 and qFDP6.3), a character that has been rarely studied (Etienne et al. 2002; Donoso et al. 2016) and for which only one QTL with major effects has been described on G4, probably because of the masking effect of this QTL on smaller QTLs in single populations. Other QTLs related to TA (qTA1, qTA2 and qTA6.2), SSC (qSSC5.1) and FW (qFW5.1 and qFW5.3) are particularly interesting because they are involved in important quality-related characters that are difficult to select for in breeding programs. The two remaining were one for MD (qMD5), which was predicted as heterozygous in seven of the peach commercial parents (Fig. 2), and one for PSC (qPSC5) segregating only in JxF. All these QTLs, along with an estimation of the genotype of the genitors used are first-hand information for the selection of parents in breeding programs.

MD and FDP are strongly correlated characters, where most of the QTLs coincide (all six MD QTLs are in positions compatible with those of six of the seven FDP QTLs), suggesting that they are the same, red skin color and SSC are characters typically influenced by the date of maturity. Thus qMD4 may be also responsible for qPSC4 and qSSC4 through pleiotropic effects of the *MD* gene.

### Breeding values

A new element from our analysis, of great interest for peach improvement and extensively developed in a companion paper [54], is the possibility of attaching a breeding value to the parents. Selecting parents with high breeding values for target characters allows for more informed decisions at the moment of cross planning. Breeding values also provide interesting information for inferring the mode of selection of different characters during peach evolution under domestication. Characters submitted to strong directional selection should present extreme breeding values in commercial vs. exotic materials. This is the case for fruit weight and red skin color, where the lowest breeding values occurred in the materials involving wild or cultivated relatives (TxE for FW and *P. davidiana* ‘P1908’ for PSC) and the highest in the modern cultivars (Nectaross for FW and JxF for PSC). In contrast, characters undergoing diversifying selection by modern breeding should exhibit a pattern where both extreme values would be found in the commercial varieties. MD and the strongly correlated FDP are typically selected by breeders to cover a broad range of maturity dates where the diversifying selection model would fit. As predicted, commercial cultivars were found to have the highest (‘Nectalady’ and ‘Belbinette’), and the lowest (‘Earlygold’) breeding values for MD and FDP. Other characters, such as SSC, TA and FD, do not have an obvious a priori pattern, of selection, For SSC, hybrids with one or both parents being exotic display the extremes of the breeding value distribution (the highest ‘BC1.25’ and the lowest ‘Weeping Flower’ (an ornamental peach) x ‘Pamirskij 5’ (a rootstock). This suggests that selection for this character in commercial breeding has been weak or ineffective, and that useful variability for breeding lines can be recovered from exotic materials. Although TA had a narrow range of breeding values, the extreme values were exhibited by commercial cultivars (‘Oro A’ for high acidity and ‘Zephyr’ for low acidity), a pattern that suggests diversifying selection. FD is a character for which one would also predict a pattern of diversifying selection, considering the broad range of geographical distribution of the peach and the need for adaptation to an ample array of chilling requirements. The extremes of the distribution of breeding values were a hybrid between two commercial varieties (JxF with GBV = 6.8) and one from a commercial and an exotic accession (PxF with GBV = −16.5), suggesting a directional selection model. This trend is confirmed by the fact that other exotic parents were also among those having the lowest breeding values, such as ‘BC1.25’ (−12.7) and TxE (−12.6). One explanation could be that, although diversifying selection occurred for FD in the commercial peach materials, the variability available for FD within this pool is low compared to that of the extended *Prunus* gene pool, where many additional alleles exist and emerge when interspecific hybrids are created. This hypothesis is moreover supported by the fact that three of the four QTLs with strong to decisive evidence for this trait (qFD4.2, qFD6.2 and qF8.2), those having the highest effects, were not detected in the set of commercial progenies analyzed.

## Conclusions

Based on the use of the FlexQTL software in a large set of families our results provide a more comprehensive view of the genetic variability of some of the most relevant agronomic characters of peach, and a deeper knowledge of their genetic basis, with the detection of many more QTLs, and identification of their effects and map positions with higher precision than with single population experiments. In addition, specific information on the parental QTL genotypes and their breeding values provides an efficient tool for parent selection in breeding programs. Our results indicate that genetic variability can come from non-commercial genotypes even for agronomic characters. Such exotic material was generally used for the introgression of resistant factors to biotic stresses in breeding programs [55] but considered as a break to recover good fruit quality. On the contrary, this study strongly highlights the potential of using exotic material to enlarge the genetic basis in the commercial cultivar pool and improve agronomic characters. In a context of breeding for sustainable orchards using fewer pesticides, introducing exotic material in selection schemes acquires a particularly important interest.

Other analyses with the same approach in *Prunus* had more modest objectives, as they were done for a single character [20] or with limited population sizes [18] but, given the deeper knowledge of the pedigrees of the materials used by these authors, these analyses could be expanded to a broader set of genotypes, some not directly involved as parents of the progenies studied. In our study, we discarded this application of the software because of the wide range of our materials and the fact that some had an uncertain pedigree. An improvement of the results reported here would be to update and complete the pedigree information using available records and completing genotyping data for a larger collection of materials using the same SNP chip used here [5]. The analysis performed also opens the door to a more integrated genetic analysis in peach, where new populations could be added to those studied here to build a more robust and complete landscape of the variability for traits of economic importance for peach and other *Prunus* crops and relatives. This would require strengthening international collaborations, such as those already proven to work satisfactorily in European and US initiatives (www.fruitbreedomics.com; www.rosbreed.org).

## Additional files

Additional file 1: Selected year of phenotypic observations per trait and progeny. (XLSX 12 kb)

Additional file 2: Distribution of the seven phenotypes analyzed. (DOCX 281 kb)

Additional file 3: Number of informative SNPs selected per progeny after filtering for data quality. (XLSX 11 kb)

## Abbreviations

BC1: Back cross 1
BC2: Back cross 2
BF: Bayes factor
C: Commercial
F1: First filial generation
F2: Second filial generation
FD: Flowering date
FDP: Fruit development period
GBV: Genome-wide bin-wise breeding value
h^2^: Narrow sense heritability
H^2^: Broad sense heritability
MD: Maturity date
NC: Non-commercial
NUI1: Nuisance variable 1
NUI2: Nuisance variable 2
PSC: Percentage of red skin overcolor
QTL: Quantitative trait loci
SNP: Single nucleotide polymorphism
SSC: Soluble solid content
STD1: Data standardization method 1
STD2: Data standardization method 2
TA: Titratable acidity
