# Supplementary material for: Integrated QTL detection for key breeding traits in multiple peach progenies
Source: BMC Genomics. 2017 Jun 6;18:404. doi: 10.1186/s12864-017-3783-6 (PMC5460339; doi:10.1186/s12864-017-3783-6)
Supplement: Supplementary file 2 — Distribution of the seven phenotypes analyzed. (DOCX 281 kb) [file 12864_2017_3783_MOESM2_ESM.docx]

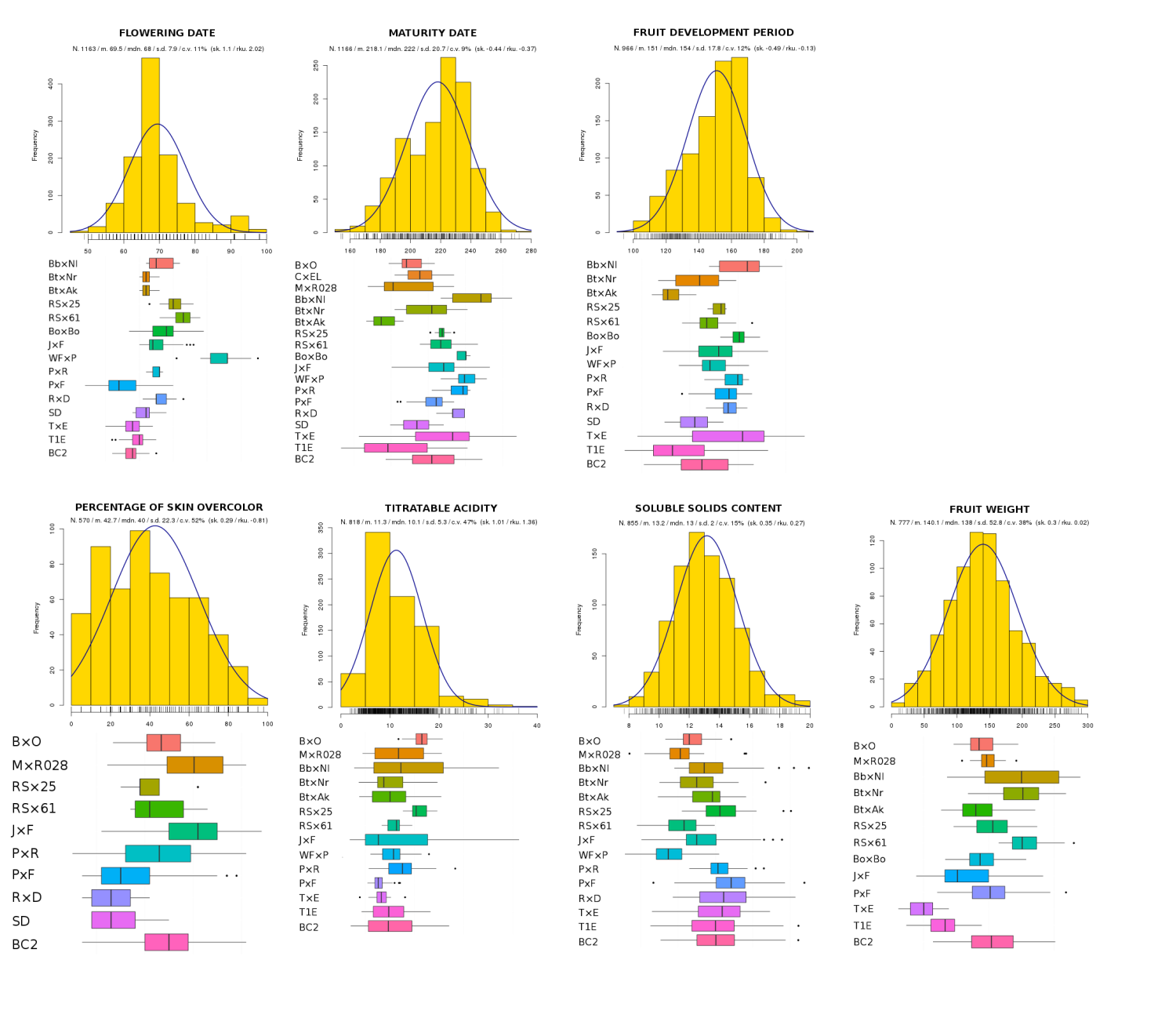
Additional file 2. Distribution of the seven phenotypes analyzed: Beginning of flowering time (day of year); Beginning of ripening time (day of year); Fruit development period (days); Percentage of red overcolor on the fruit skin (surface percentage); Titrable acidity (meq/100ml); Soluble solid content (brix degrees); Weight of the whole fruit (grams). The number of individuals (N), means (m), medians (mdn), standard deviations (sd), coefficients of variation (cv), skewness (sk) and kurtosis (rku) of the distributions are indicated underneath each trait name. Histograms show the distribution for all the individuals from the different progenies. The blue lines correspond to the normal distributions with the same mean and standard deviation obtained for each phenotype. The distribution of the phenotype values for each progeny is also shown on the boxplots below histograms.
